# Internalized Stigma Is a Predictor of Mental Health Secrecy and Loneliness in Young People With Clinical Depression Symptoms: A Longitudinal Study

**DOI:** 10.1002/jclp.23789

**Published:** 2025-03-26

**Authors:** Katie Prizeman, Ciara McCabe, Netta Weinstein

**Affiliations:** ^1^ Department of Psychology and Clinical Language Sciences University of Reading Reading UK

**Keywords:** affective disorders, chronic mental illness, clinical depression, internalized stigma, loneliness, longitudinal, mental health secrecy

## Abstract

Young people with depression experience loneliness and internalized stigma. Stigma might make disclosing depression to others difficult, thus increasing loneliness and reducing the opportunity for treatment. Knowing whether internalized stigma predicts loneliness and secrecy reinforces the need for stigma reduction efforts. The aim of this research was to examine the independent effects of internalized stigma and clinical depression on loneliness and mental health secrecy in young people with a range of depressive symptoms (Mood and Feelings Questionnaire score ≥ 27). A total of 275 young people (*M*_age_ = 20.53, SD = 2.17) were recruited and completed the Internalized Stigma of Mental Illness Inventory, the 5‐Item Link's Secrecy Scale, and the UCLA Loneliness Scale at baseline and again at 1‐month follow‐up (*N* = 172, *M*_age_ = 20.40, SD = 2.00). Results showed that internalized stigma was associated with baseline loneliness (*β* = 0.57, 95% CI: 7.87–11.75, *p* < 0.001), baseline secrecy (*β* = 0.40, 95% CI: 0.23–0.45, *p* < 0.001), and secrecy over time (*β* = 0.20, 95% CI: 0.04–0.30, *p* = 0.009). This work highlights the need to develop targeted interventions to reduce stigma and encourage mental health disclosure and help‐seeking behaviors among young people with depression.

**Abbreviations**
ISMI‐9*: Internalized Stigma of Mental Illness Inventory—9‐Item Version
M: mean
MDD: major depressive disorder
MFQ: Mood and Feelings Questionnaire
SD: standard deviation
T1: Time 1
T2: Time 2
UCLA Loneliness Scale: University of California, Los Angeles Loneliness Scale

## 1 Introduction

Major depressive disorder (MDD) is the main cause of disease and disability among young people worldwide (Achterbergh et al. 2020), with over 40% of first episodes occurring before the age of 20 (Malhi and Mann 2018). Early intervention programs for MDD are being implemented more widely around the world. Research shows that psychological prevention methods can be effective and may offer significant therapeutic benefits, particularly for young people who are at risk of developing depression (Beames et al. 2021; Stockings et al. 2016; van Zoonen et al. 2014; Werner‐Seidler et al. 2017).

Depression is considered a major risk factor for loneliness (Cacioppo et al. 2006, 2014; Cornwell and Waite 2009), the negative emotional response to perceived social separateness, when one feels a difference between the desired and perceived quality of one's social relationships (Badcock et al. 2016; Cacioppo et al. 2006). Loneliness is linked to numerous negative outcomes, not least suicide (Chen et al. 2023; Shoib et al. 2023). The risk of depressed individuals feeling lonely may be heightened during the period between adolescence and early adulthood, when both friendships and romantic relationships are seen as especially important (Hawthorne 2008; Qualter et al. 2015; Victor and Yang 2012). Although the prevalence of loneliness varies with age, its connection to depression remains constant across the lifespan (Barreto et al. 2022; Nolen‐Hoeksema and Ahrens 2002). Knowing how to decrease loneliness could lead to improved mental health outcomes.

Apart from the direct challenges that depressive symptoms pose to young people, societal acceptance of mental health conditions presents an indirect challenge. Labeling or self‐labeling as “mentally ill” may result from early intervention itself as well as indications of the developing illness (Yang et al. 2010). Specifically, those with depression are met with stigma—others' negative societal views, including stereotypes, prejudices, and discriminatory behaviors (Corrigan et al. 2005; Corrigan and Watson 2002; DeLuca 2020)—concerning their mental health that extends to global judgments of them as individuals (Prizeman et al. 2023; Reavley et al. 2018). There are two main types of stigma related to mental health: (1) public stigma, which occurs when others treat individuals with mental health conditions, such as depression, in a discriminatory way, and (2) internalized stigma, where people with mental health conditions adopt stigmatizing beliefs and expect their diagnosis to be associated with negative labels (Corrigan et al. 2005; Corrigan and Watson 2002).

Regardless of whether clinical symptoms are present, internalized stigma can further exacerbate mental health challenges and negatively impact the wellbeing of young people (Corrigan and Watson 2002; DeLuca 2020; Kaushik et al. 2016; Yang et al. 2010). Past research has suggested young people with depression are typically more prone to experiencing stigma (Mukolo et al. 2010) and its long‐lasting negative effects (Buchholz et al. 2015; Kranke et al. 2011; Rüsch, Brohan et al. 2014; Rüsch, Müller et al. 2014). Cross‐sectional research has identified associations between public stigma, shame, and self‐labeling with increased stigma‐related stress, and between higher levels of stigma stress and reduced wellbeing (Rüsch, Brohan et al. 2014; Rüsch, Müller et al. 2014; Switaj et al. 2014). These experiences, along with the negative self‐perceptions that often accompany them, can have profound long‐term effects on future health and create developmental challenges throughout adulthood (Hertzman and Boyce 2010; Liggins and Hatcher 2005; Matthews et al. 2016; Prizeman et al. 2023; Steinberg 2005). They can also lead to chronic stress, heightened feelings of loneliness, victimization, rejection, social isolation, withdrawal, mental health secrecy, and devaluation (Ferrie et al. 2020; Mannarini and Rossi 2018; Prizeman, McCabe et al. 2024).

We explore the possibility that stigma may be linked to increased feelings of loneliness, which are commonly associated with depression (Matthews et al. 2016; Paskaleva‐Yankova 2022). For instance, stigma can influence social behaviors, heightening fears of social rejection, judgment, and negative labeling (Oexle et al. 2017). As a result, stigmatized youth may hide or fail to disclose their mental health condition (Lasalvia et al. 2013; Prizeman, McCabe et al. 2024; Prizeman, Weinstein et al. 2024; Rüsch, Brohan et al. 2014; Rüsch, Müller et al. 2014), leading them to avoid social interactions (Mayer et al. 2022). In turn, the lack of social engagement increases the likelihood of experiencing adverse emotional outcomes, such as feelings of loneliness (Badcock et al. 2016; Cacioppo et al. 2006).

Disclosure of depression is not an easy task. For individuals with depression, coping with their condition and discussing it with others can make social connections more challenging (Prizeman et al. 2023; Prizeman, McCabe et al. 2024; Prizeman, Weinstein et al. 2024; Rüsch et al. 2005; Schomerus et al. 2012; Wahl 1999). Disclosure is thought to have long‐term detrimental effects, including stigma and feelings of loneliness (Corrigan et al. 2010; Mayer et al. 2022; Prizeman et al. 2023; Prizeman, McCabe et al. 2024; Thornicroft et al. 2022). While mental health secrecy may temporarily protect individuals from stigma, it can also lead to negative outcomes, such as loneliness, social isolation, and a decline in overall wellbeing (Mayer et al. 2022; Pachankis 2007; Prizeman, McCabe et al. 2024; Switaj et al. 2014). Secrecy is often considered a behavioral expression of internalized stigma, though the two constructs can be conceptually distinguished. Internalized stigma refers to the adoption of negative societal beliefs about mental health, which may lead individuals to conceal their struggles to avoid discrimination or rejection. While secrecy is inherently multidimensional, our study focuses specifically on how mental health stigma drives the concealment of mental health issues and the behaviors associated with it. This encompasses both overt actions, such as withholding information about one's mental health, and more subtle, internalized behaviors, such as avoiding situations where mental health challenges might be revealed (Link et al. 1991).

Past research has primarily focused on (1) public stigma; (2) adults and older people with mental health problems; or (3) young people with other mental illnesses, such as psychosis or autism, but not specifically depression (Depla et al. 2005; Earnshaw and Quinn 2012; Rüsch, Brohan et al. 2014; Rüsch, Müller et al. 2014). There is a lack of longitudinal quantitative data on how internalized stigma, combined with depression symptoms in young people, affects loneliness and mental health secrecy over time. However, internalized stigma may offer a more comprehensive explanation for the loneliness and mental health secrecy experienced by depressed young people than depression symptoms alone. This paucity of knowledge may undermine efforts to build informed interventions to help young people with depression reconnect and reduce the stigma and secrecy surrounding mental health. Addressing this gap will enable the development of more targeted strategies to mitigate stigma, enhance mental health outcomes, and facilitate open dialogue, ultimately reducing isolation and fostering greater social connection among young people.

This study aimed to assess whether internalized stigma above and beyond clinical depression symptoms drives subsequent loneliness and mental health secrecy in young people. We hypothesized that clinical depression and internalized stigma would be associated with loneliness and mental health secrecy at baseline and over time. Our hypotheses were: (1) Clinical depression would predict loneliness and mental health secrecy at baseline, and these associations would persist over time, among young people with a range of clinical depressive symptoms; and (2) Internalized stigma would predict loneliness and mental health secrecy at baseline, with these associations likely to persist at 1‐month follow‐up, even after controlling for baseline loneliness and secrecy, in young people with a range of clinical depressive symptoms.

## 2 Transparency and Openness

### 2.1 Data, Materials, Code, and Online Resources

Deidentified data are publicly available and can be accessed through the University of Reading's Research Data Archive. We report how we determined our sample size, data exclusions, and all measures in the study.

### 2.2 Ethical Considerations

Study procedures were initially approved by the University Research Ethics Committee (2022‐072‐NW) of the University of Reading. The authors assert that all procedures contributing to this work comply with the ethical standards of the relevant national and institutional committees on human experimentation and with the Helsinki Declaration of 1975, as revised in 2008, and that written informed consent was given.

## 3 Methods

### 3.1 Context and Purpose of the Research

This study investigates how internalized stigma and clinical depression independently influence loneliness and secrecy over time in young people aged 17–25 with a range of depressive symptoms, as indicated by a score > 27 on the Mood and Feelings Questionnaire (MFQ). The data collected were exclusively for this study and were not used for any other purpose, ensuring that the research questions were addressed with data specifically gathered to explore these relationships.

### 3.2 Sample Size and Exclusions

The sample size was determined based on statistical power calculations to ensure sufficient power for detecting associations between stigma, loneliness, and secrecy. Participants were included if they met the depression symptom criteria (MFQ ≥ 27). Exclusions were made for incomplete data at either time point or if participants did not meet the clinical depressives symptom threshold.

#### 3.2.1 Power

We conducted a priori G*Power analysis to calculate the minimum sample size required for the present study. The analysis was based on a linear multiple regression: fixed model, single regression coefficient (*t* tests), with four predictors, as we were interested in examining the relationships between internalized stigma, clinical depression, loneliness, and mental health secrecy. We set the parameters for the analysis with an acceptable margin of error of 5%, a power of 0.95, an *α* value of 0.05 (*α* = 0.05), and an effect size of *f* ^2^ = 0.1, which was informed by similar studies in the field (Coutts‐Smith and Phillips 2023). The results indicated that a sample size of 132 participants would be sufficient to detect meaningful effects in the study.

### 3.3 Participants and Recruitment

Young people (*N* = 275), aged 17–25 (*M*_age_ = 20.53, SD = 2.17), with clinical levels of depressive symptoms [MFQ—a score of ≥ 27] (Costello and Angold 1988), were recruited from local schools and the student population via the School of Psychology research panel, online advertisements, and posters.

Participants were reimbursed for their time and effort by being entered into a draw for a £50 Amazon voucher. Participants who consented to take part in the follow‐up phase were contacted via email ~1 month after the initial data collection. To reimburse participants for their effort at follow‐up, we entered participants into a further draw for one of five £50 Amazon vouchers.

#### 3.3.1 Participant Dropout and Attrition at Follow‐Up (*N* = 172)

The reduced sample size at follow‐up in this study was primarily due to two factors: participant attrition and nonresponse. First, some participants withdrew from the study, although the reasons for their withdrawal were unknown. Second, a number of participants who completed the baseline assessment could not be reached for the follow‐up. Despite the reduction in sample size, demographic characteristics and mean values for key variables remained similar between baseline and follow‐up, although participants at follow‐up had, on average, lower depressive scores. See Table 1. Importantly, all participants who completed the follow‐up assessment met the initial inclusion criteria, with depression scores > 27 on the MFQ, indicating clinically significant depression.

**Table 1:** Participant sociodemographic and descriptive characteristics at Time 1 (*N* = 275) and Time 2 (*N* = 172).

	Mean (SD)			
Descriptive characteristics	T1	T2		Range
Age	20.53 (2.17)	20.40 (2.00)		17–25
Clinical depression (MFQ)	38.75 (9.15)	27.26 (14.16)		0–66
Internalized stigma (ISMI‐9*)	2.21 (0.64)	2.16 (0.65)		1–4
Secrecy (5‐Item Link's Secrecy Scale)	3.57 (0.53)	3.50 (0.53)		1–6
Loneliness (UCLA)	51.25 (11.01)	49.67 (11.96)		0–80

**Demographic characteristics**	***N*** **(%)**			
	**T1**		**T2**	
Gender				
Female	213 (77.45)		136 (79.07)	
Male	52 (18.91)		31 (18.02)	
Other	7 (2.55)		4 (2.33)	
Prefer not to say	3 (1.09)		1 (0.58)	
Ethnicity				
Asian or Asian British	50 (18.18)		34 (19.77)	
Arabic	10 (3.64)		6 (3.49)	
Black/African American	17 (6.18)		11 (6.40)	
Hispanic/Latino/Spanish	5 (1.82)		3 (1.74)	
White	163 (59.27)		106 (61.63)	
Mixed/Multiple ethnic background	25 (9.09)		12 (6.98)	
Other racial‐ethnic group	5 (1.82)		—	
Country				
India	7 (2.55)		5 (2.91)	
Kenya	3 (1.09)		2 (1.16)	
Malaysia	8 (2.91)		5 (2.91)	
South Africa	19 (6.91)		10 (5.81)	
United Kingdom	205 (74.55)		135 (78.49)	
United States	18 (6.55)		5 (2.91)	
Other (> 1%)	15 (5.45)		10 (5.81)	
Education level				
High school	22 (8.00)		9 (5.23)	
University	253 (92.00)		163 (94.77)	

### 3.4 Procedure

Participants received a link to the online information sheet and consent form. After reading the information sheet, participants were provided the opportunity to ask questions about the study via email. After giving written consent, they completed the measures described below via a Jisc online survey platform. All study information and consent procedures were conducted at the outset of the research, and no additional consent was required for the follow‐up assessment.

## 4 Data Collection

### 4.1 Demographics

All participants completed demographic questions about age, gender, education, and ethnicity at baseline.

### 4.2 Measures

Data were collected at both Time 1 (T1) and Time 2 (T2) for all measures included in the study.

#### 4.2.1 Mood and Feelings Questionnaire (MFQ)

Participants completed the MFQ (Costello and Angold 1988) as a prescreening for depressive symptoms before taking part in the study. The MFQ is a 33‐item scale that measures depressive symptoms, has been validated in clinical trials, and is suitable for adolescents (Burleson Daviss et al. 2006; Jarbin et al. 2020). Responses indicate how they have been feeling or acting in the past 2 weeks (Costello and Angold 1988); high scores indicate greater depressive symptoms. Each item is rated on a 3‐point scale from 0 (*not true*), 1 (*somewhat true*), to 2 (*true*). Total scores, which range from 0 to 66, were determined by the sum of all items. The scale demonstrates excellent internal reliability (Cronbach's *α* = 0.91–0.93) and sufficient validity, with a recommended cut‐off score of 27 for distinguishing clinical from nonclinical populations (Thabrew et al. 2018). This cut‐off point provides the best diagnostic confidence, as determined by the intersection of sensitivity [0.78 (95% CI: 0.67–0.89)] and specificity [0.78 (95% CI: 0.66–0.89)], as reported by Wood et al. (1995). A score of 27 or higher is also indicative of clinically significant depression (Wood et al. 1995). Example items include, “I felt miserable or unhappy,” “I didn't enjoy anything at all,” “I thought that life wasn't worth living,” and “I found it hard to think properly or concentrate.” This questionnaire is widely used to score depression in young people (Wood et al. 1995).

#### 4.2.2 Internalized Stigma of Mental Illness Inventory—9‐Item Version (ISMI‐9*)

The ISMI‐9* scale is a 9‐item self‐report questionnaire that produces a total score ranging from 1 to 4. Each item is rated on a 4‐point scale from 1 (*strongly disagree*), 2 (*disagree*), 3 (*agree*), to 4 (*strongly agree*). Scores range from 1 (*minimal to no internalized stigma*) to 4 (*severe internalized stigma*; that is, higher scores indicated more internalized stigma) (Boyd et al. 2014) and has strong internal consistency (Cronbach's *α* = 0.86) (van Beukering et al. 2022). Example items include, “Stereotypes about the mentally ill apply to me,” “People without mental illness could not possibly understand me,” and “I can't contribute anything to society because I have a mental illness.”

#### 4.2.3 Link's Secrecy Scale

The 5‐Item Link's Secrecy Scale, developed by Link et al. (1991), measures an individual's tendency to conceal mental health conditions. Participants rate 29 items on a 6‐point scale (1 = *strongly disagree*; 6 = *strongly agree*), with higher scores indicating greater secrecy. The scale was designed to assess a single dimension of mental health secrecy, but it also captures various aspects of concealment, including the avoidance of help‐seeking and hiding mental health struggles from social circles, such as family and friends. While some items reflect participants' perceptions of others' reactions to mental health disclosure, the scale primarily focuses on the individual's tendency to conceal their mental health conditions. Although the scale has not been extensively tested through factor analysis, it has shown strong internal consistency, with Cronbach's *α* values ranging from 0.70 to 0.85 across studies, providing support for its reliability and validity. Example items include, “A former mental patient will have to disguise his or her past hospitalization to acquire a job” and “If I had a close relative who had been treated for a serious mental illness, I would advise him or her not to tell anyone about it.”

#### 4.2.4 UCLA Loneliness Scale (UCLA)

The UCLA Loneliness Scale is a 20‐item general measure of loneliness, with responses graded on a 4‐point rating scale (1 = *never*; 2 = *rarely*; 3 = *sometimes*; 4 = *often*). Scores range from 0 to 80. It is found to have satisfactory psychometric properties and satisfactory reliability and factorial validity (Cronbach's *α* = 0.89–0.94) (Russell 1996). Example items include “How often do you feel that you lack companionship?,” and “How often do you feel that your relationships with others are not meaningful?”. Higher scores indicated more loneliness (Link et al. 1991; Russell et al. 1978).

### 4.3 Data Analyses

Data were analyzed using the latest version of IBM SPSS Statistics (Version 29).

**Figure 1:**
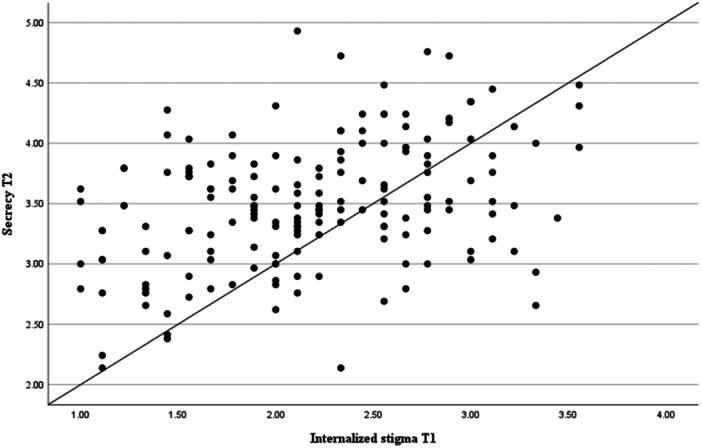
Scatter plot showing the relationship between internalized stigma at Time 1 and secrecy at Time 2.

We conducted Pearson's correlations (*r*) to assess linear relationships between all continuous variables at baseline and follow‐up. See Table 2. We used multiple linear regression analyses to examine the relationships between the variables at baseline and over time. See Tables 3, 4 and Figure 1.

**Table 2:** Bivariate correlational analyses.

Scales	1	2	3	4	5	6
1. Clinical depression T1	—					
2. Internalized stigma T1	0.57**	—				
3. Loneliness T1	0.44**	0.63**	—			
4. Secrecy T1	0.30**	0.44**	0.26**	—		
5. Loneliness T2	0.37**	0.53**	0.81**	0.25**	—	
6. Secrecy T2	0.19*	0.38**	0.20**	0.60**	0.32**	—

Abbreviations: T1 = Time 1, T2 = Time 2.

* *p* < 0.05

** *p* < 0.01.

**Table 3:** Multiple regression analyses predicting loneliness and secrecy at Time 1 (*N* = 275) from predictor variables clinical depression and internalized stigma at Time 1.

	Loneliness T1					Secrecy T1				
*Predictors T1*	*β*	se	*t*	*p*	*R* ^2^	*β*	se	*t*	*p*	*R* ^2^
Clinical depression	0.12	0.07	2.03	0.043	0.41	0.07	0.00	1.03	0.303	0.20
Internalized stigma	0.60	0.99	9.94	< 0.001	0.41	0.40	0.06	6.01	< 0.001	0.20

*Note:* Model. Clinical depression T1 and internalized stigma T1 scores were run in the same model when predicting loneliness T1 and secrecy T1. Baseline sample size: *N* = 275. Control variables: None (there are no additional variables in these models that would serve as controls). Outcome variables: Loneliness T1 and secrecy T1 (listed in the top column). Predictor variables: Clinical depression T1, internalized stigma T1, loneliness T1, and secrecy T1 (listed in the left‐hand column). *R* ^2^ values represent the proportion of variance in the dependent variable explained by the independent variables.

Abbreviation: T1 = Time 1.

**Table 4:** Multiple regression analyses predicting loneliness and secrecy at Time 2 (*N* = 172) from predictor variables clinical depression, internalized stigma, loneliness, and secrecy at Time 1 (*N* = 275).

	Loneliness T2					Secrecy T2				
*Predictors T1*	*β*	se	*t*	*p*	*R* ^2^	*β*	se	*t*	*p*	*R* ^2^
Clinical depression	0.01	0.07	0.24	0.814	0.66	−0.07	0.00	−1.92	0.358	0.39
Internalized stigma	0.04	1.20	0.66	0.511	0.66	0.20	0.06	2.64	0.009	0.39
Loneliness	0.78	0.06	13.49	< 0.001	0.66	—	—	—	—	—
Secrecy	—	—	—	—	—	0.54	0.06	8.23	< 0.001	0.39

*Note:* Models. Clinical depression T1, internalized stigma T1, and loneliness T1 scores were run in the same model when predicting loneliness T2. Clinical depression T1, internalized stigma T1, and secrecy T1 scores were run in the same model when predicting secrecy T2. Baseline sample size: *N* = 275; Follow‐up sample size: *N* = 172. Control variables: Loneliness T1 and secrecy T1 (included as independent variables alongside clinical depression and internalized stigma T1). Outcome variables: Loneliness T2 and secrecy T2 (listed in the top column). Predictor variables: Clinical depression T1, internalized stigma T1, loneliness T1, and secrecy T1 (listed in the left‐hand column). *R* ^2^ values represent the proportion of variance in the dependent variable explained by the independent variables.

Abbreviations: T1 = Time 1, T2 = Time 2.

## 5 Results

### 5.1 Demographics

Participant demographic and descriptive characteristics are presented in Table 1.

### 5.2 Correlational Analyses

#### 5.2.1 Relationships Between Internalized Stigma (ISMI‐9*), Clinical Depression (MFQ), Loneliness (UCLA), and Secrecy (Secrecy Scale)

We used Pearson's (*r*) to examine the correlation between clinical depression (MFQ) and internalized stigma (ISMI‐9*) scores at T1, and secrecy (Secrecy Scale) and loneliness (UCLA) scores at T1 and at T2. See Table 2.

## 6 Longitudinal Analyses

### 6.1 Multiple Linear Regressions at T1

#### 6.1.1 Relationship Between Internalized Stigma (ISMI‐9*), Clinical Depression (MFQ), and Loneliness (UCLA) at T1

Internalized stigma (*β* = 0.57 (95% CI: 7.87–11.75), *p* < 0.001) and clinical depression (*β* = 0.12 (95% CI: 0.00–0.27), *p* = 0.043) were significant predictors of loneliness at T1. See Table 3.

#### 6.1.2 Relationship Between Internalized Stigma (ISMI‐9*), Clinical Depression (MFQ), and Secrecy (Secrecy Scale) at T1

Internalized stigma significantly predicted secrecy at T1 (*β* = 0.40 (95% CI: 0.23–0.45), *p* < 0.001) and clinical depression did not predict secrecy at T1 (*β* = 0.07 (95% CI: −0.00 to 0.01), *p* = 0.303). See Table 3.

### 6.2 Multiple Linear Regressions With T1 Predicting T2 Outcomes

#### 6.2.1 The Association Between Internalized Stigma (ISMI‐9*), Clinical Depression (MFQ), and Loneliness (UCLA) at T1, and Loneliness (UCLA) at T2

Loneliness at T1 predicted loneliness at T2 (*β* = 0.78 (95% CI: 0.71–0.95), *p* < 0.001). Internalized stigma at T1 (*β* = 0.04 (95% CI: −1.58 to 3.17), *p* = 0.511), and clinical depression at T1 (*β* = 0.01 (95% CI: −0.13 to 0.16), *p* = 0.814) did not predict loneliness T2 when controlled for loneliness T1. See Table 4.

#### 6.2.2 The Association Between Internalized Stigma (ISMI‐9*), Clinical Depression (MFQ), and Secrecy (Secrecy Scale) at T1, and Secrecy (Secrecy Scale) at T2

Secrecy at T1 predicted secrecy at T2 (*β* = 0.54 (95% CI: 0.39–0.64), *p* < 0.001), and internalized stigma at T1 predicted secrecy at T2 (*β* = 0.20 (95% CI: 0.04–0.30), *p* = 0.009). Clinical depression at T1 (*β* = −0.07 (95% CI: −0.01 to 0.01), *p* = 0.358) did not predict secrecy at T2 when we controlled for secrecy at T1. See Table 4 and Figure 1.

## 7 Discussion

The present study aimed to explore the relationships between clinical depression, internalized stigma, loneliness, and mental health secrecy. Our hypotheses were: (1) Clinical depression would predict loneliness and mental health secrecy at baseline, and these associations would persist over time, among young people with a range of clinical depression symptoms; and (2) Internalized stigma would predict loneliness and mental health secrecy at baseline, with these associations likely to persist at 1‐month follow‐up, even after controlling for baseline loneliness and secrecy, in young people with a range of clinical depression symptoms.

Our correlation analyses supported these hypotheses, revealing positive associations between internalized stigma, clinical depression, mental health secrecy, and loneliness at both baseline and follow‐up. Specifically, we found that both internalized stigma and clinical depression were independently associated with loneliness. However, it was internalized stigma, rather than clinical depression, that was uniquely linked to increased mental health secrecy in young people. Over time, internalized stigma contributed to further changes in mental health secrecy, suggesting its continued influence.

These findings build on previous research (Antonelli‐Salgado et al. 2021; Gulliver et al. 2010; Prizeman et al. 2023), which emphasizes the significant role of mental health stigma in hindering wellbeing. Our study builds on this research by focusing on youth with depressive symptoms, which often co‐occur with stigma, to predict outcomes related to loneliness and secrecy. Even after controlling for loneliness and secrecy, internalized stigma remained a significant predictor of both outcomes, highlighting the critical role of stigma as a barrier to mental health recovery in young people (Prizeman, Weinstein et al. 2024).

The use of longitudinal data allowed us to examine how internalized stigma and clinical depression impacted loneliness and secrecy over time. The longitudinal analyses revealed consistent associations between internalized stigma and the effects across the study period. Specifically, when predicting secrecy 1 month later, internalized stigma—rather than clinical depressive symptoms—accounted for significant variance. In other words, our findings indicated that internalized stigma, rather than clinical depression itself, influenced young people's secrecy. Moreover, stigma was associated with further changes in secrecy scores, while the results did not show a significant relationship between depression and secrecy in this sample. These findings suggest that internalized stigma plays a key role in shaping secrecy, more so than clinical depressive symptoms alone. Our results align with previous studies indicating that individuals with mental health conditions, as a marginalized group, may experience lower wellbeing, reduced quality of life, and greater challenges in recovery (Chan and Tsui 2023; Divin et al. 2018; Mejia‐Lancheros et al. 2021; Pérez‐Garín et al. 2017; Rickwood et al. 2005; Villatoro et al. 2022; Yap et al. 2011; Yip et al. 2023). For example, mental health symptoms such as guilt and self‐blame can increase stigma, which may prevent young people from disclosing their mental health (i.e., increase mental health secrecy), which may have negative effects such as loneliness and social isolation (Oexle et al. 2018; Pachankis 2007; Prizeman et al. 2023; Prizeman, McCabe et al. 2024; Prizeman, Weinstein et al. 2024). One possible explanation is that younger individuals may experience higher rates of social exclusion and isolation (Sawyer et al. 2012). This could be due to various factors, such as challenges related to social identity, the stigma of mental health conditions, or difficulties in forming supportive relationships and accessing social networks during this transitional period of life. More research is needed to compare the social experiences and stigma‐related challenges faced by younger and older individuals with mental health conditions. This would help identify age‐specific barriers to supporting and treating young people with depression. Understanding how stigma affects young people differently could lead to tailored interventions and more effective strategies for improving mental health outcomes (Prizeman, Weinstein et al. 2024; Rickwood et al. 2005; Sawyer et al. 2001).

Past research has shown that young people who disclose their mental health condition receive support, experience acceptance, and receive fewer stigmatizing responses (Bril‐Barniv et al. 2017; Prizeman, McCabe et al. 2024; Rüsch et al. 2019). This can make the decision to disclose one's mental illness easier and can improve quality of life, enhance psychological growth, and increase help‐seeking (Corrigan et al. 2010; Rüsch, Brohan et al. 2014; Rüsch, Müller et al. 2014). In turn, it may lessen feelings of loneliness, social isolation, and withdrawal (Prizeman, McCabe et al. 2024), all of which improve the recovery process (Bril‐Barniv et al. 2017). Building on the findings of the current study, future research should aim to examine how stigma‐reduction strategies can be incorporated into mental health treatments to improve outcomes for young people with depression.

Also, in keeping with prior studies, our results showed that young people with higher internalized stigma scores were more likely to be lonely. Loneliness has been linked to a fear of being judged inadequate and a fear of rejection (Achterbergh et al. 2020; Antonelli‐Salgado et al. 2021; Cacioppo et al. 2006; Hards et al. 2022; Watson and Nesdale 2012). Moreover, loneliness and depression may result in a vicious cycle for young people (Elmer and Stadtfeld 2020; Hards et al. 2022; Prizeman, McCabe et al. 2024). Young people who feel lonely might thus withdraw from social interactions, which in turn can exacerbate their depression. Similarly, those with a depression diagnosis and/or symptoms often socially isolate themselves and have a disconnection from society (that stems from internalized stigma), leading to feelings of loneliness as well as shame and low self‐esteem (Gadassi and Rafaeli 2015; Niu et al. 2022; Prizeman et al. 2023; Prizeman, McCabe et al. 2024; Segrin 2000). Given its role in youth depression, alleviating loneliness is an important and cost‐effective public health intervention for this population.

Taken together, the results of the study suggest that the experience of internalized stigma exists for young people in relation to mental health. Stigma toward mental disorders is one of the main barriers to help‐seeking and access to mental health services for young people worldwide (Antonelli‐Salgado et al. 2021; Prizeman et al. 2023). Young people with mental health conditions face significant risks to their self‐esteem, self‐efficacy, and interpersonal connections due to stigma‐related factors such as preconceptions, prejudices, and discrimination. These experiences can contribute to feelings of fear, loneliness, and social isolation, which may lead young people to keep their mental health struggles hidden in order to avoid negative consequences.

## 8 Strengths, Limitations, and Future Directions

This study offers important insights into the complex relationships between internalized stigma, loneliness, and secrecy among young people with depression. A notable strength of the study is the use of a well‐defined sample (*N* = 275), with a focus on young individuals who varied in the severity of their clinical depressive symptoms. The inclusion of several validated measures—such as the MFQ, the ISMI‐9, the Link's Secrecy Scale, and the UCLA Loneliness Scale—enhances the reliability and validity of the constructs assessed. Additionally, the study's longitudinal design, with a 1‐month follow‐up, provides valuable insight into the temporal effects of internalized stigma on both loneliness and secrecy, shedding light on how stigma may influence these outcomes over time. The findings also offer robust statistical support (with significant effect sizes) for the hypothesis that internalized stigma is independently associated with higher levels of loneliness and secrecy, highlighting the need for stigma reduction for youth depression. Another strength of the study is its statistical control for loneliness and secrecy at T1 when predicting loneliness and secrecy at T2. By examining the independent effects of internalized stigma, the study strengthens the evidence for a direct relationship between stigma and the psychological outcomes of loneliness and secrecy, independent of depression symptom severity.

Despite these strengths, several limitations should be considered when interpreting the results. First, the study relies on self‐reported data, which can be subject to biases such as social desirability or recall bias. This is particularly relevant given the sensitive nature of the topics (depression, loneliness, and stigma). Future research could benefit from incorporating more objective measures or triangulating self‐reports with clinical observations or behavioral indicators. Second, although the sample included a range of depression symptoms, it was limited to young people aged 17–25, which may reduce the generalizability of the findings to other age groups or populations with different social or cultural contexts. Additionally, while the study examines the effects of internalized stigma, it does not explore potential mediators or moderators, such as social support, coping strategies, or cultural factors, which could influence the relationship between stigma, loneliness, and secrecy. Future studies could address these gaps by exploring these factors and using a more diverse sample. Another limitation of the study is that the 5‐Item Link's Secrecy Scale may conflate secrecy with internalized stigma, as the two constructs are closely related and sometimes difficult to separate. This overlap does not invalidate the scale, but it highlights the need for future research to more clearly distinguish between secrecy and stigma. Understanding whether secrecy is an independent construct, or a direct result of internalized stigma could provide important insights into how stigma affects help‐seeking behavior and mental health outcomes. Future studies should explore this relationship further, as doing so would help clarify the role of secrecy in mental health struggles and its potential to predict negative outcomes, such as social isolation and reduced treatment engagement. Finally, the potential priming effect of using stigma‐focused scales like the ISMI‐9*, which explicitly mentions “mental illness.” This could lead participants to focus more on the stigma of mental health, possibly influencing their responses. Although the ISMI‐9* is a validated tool for measuring internalized stigma, this priming effect may narrow the understanding of stigma by making mental illness the central focus. Future research should explore whether this priming effect impacts responses and whether alternative or revised measures of stigma could reduce this bias.

Future research on stigma could play a crucial role in improving how loneliness and mental health secrecy are addressed in treatments. By identifying effective stigma‐reduction strategies, future studies could help integrate interventions that not only lessen the harmful effects of stigma but also address the secrecy and loneliness that contribute to social isolation. For example, research could explore how reducing stigma around mental health might encourage individuals to be more open about their struggles, thereby reducing secrecy and fostering greater social connections. This openness could improve engagement in therapies like cognitive‐behavioral therapy or interpersonal therapy, both of which aim to alleviate loneliness by improving social relationships. Furthermore, incorporating stigma‐reduction strategies into group therapy could provide a supportive environment where shared experiences of overcoming stigma and secrecy help build a sense of community, ultimately reducing loneliness and lowering the barriers to seeking help among young people.

## 9 Conclusion

In conclusion, we emphasize the importance of reducing stigma among young people to reduce the loneliness and secrecy associated with depression. By examining how internalized stigma and clinical depression contribute to loneliness and mental health secrecy over time, our research has found the stigma associated with depression leads to secrecy, thus providing a more nuanced understanding of the challenges faced by young people with depression. While mental health secrecy may temporarily safeguard people from stigma, it can also worsen negative effects such as loneliness, social isolation, and wellbeing.

This knowledge is, therefore, essential for future studies to examine the best possible ways to reduce internalized stigma and design interventions that not only address depression but also tackle the stigma and secrecy that often prevent young people from seeking help and building supportive relationships. Ultimately, this research could lead to more effective, tailored strategies to minimize stigma, improve mental health outcomes, reduce loneliness and social isolation, and promote open conversations about mental health in this population.

## Author Contributions

Conceptualization and study design: Katie Prizeman, Netta Weinstein, and Ciara McCabe. Data curation: Katie Prizeman. Formal analysis: Katie Prizeman. Investigation: Katie Prizeman. Methodology: Katie Prizeman, Netta Weinstein, and Ciara McCabe. Project administration: Katie Prizeman. Resources: Katie Prizeman. Software: Katie Prizeman. Supervision: Katie Prizeman, Netta Weinstein and Ciara McCabe. Validation: Katie Prizeman. Visualization: Katie Prizeman, Netta Weinstein, and Ciara McCabe. Writing – original draft: Katie Prizeman. Writing – reviewing and editing: Katie Prizeman, Netta Weinstein, and Ciara McCabe. All authors read and approved the final manuscript.

## Ethics Statement

The study was approved by the University Research Ethics Committee (2022‐072‐NW) of the University of Reading. The authors assert that all procedures contributing to this work comply with the ethical standards of the relevant national and institutional committees on human experimentation and with the Helsinki Declaration of 1975, as revised in 2008.

## Consent

Informed consent was obtained from all subjects involved in the study.

## Conflicts of Interest

The authors declare no conflicts of interest.

## Data Availability

Deidentified data are publicly available and can be accessed through the University of Reading's Research Data Archive. Prizeman, Katie (2024): Data supporting: “Internalized stigma is a predictor of mental health secrecy and loneliness in young people with depression symptoms: a longitudinal study.” University of Reading. Data set. https://doi.org/10.17864/1947.001318.
